# Implementation and dissemination of the *Sikh American Families Oral Health Promotion Program*

**DOI:** 10.1007/s13142-017-0466-4

**Published:** 2017-01-31

**Authors:** Mary E. Northridge, Rucha Kavathe, Jennifer Zanowiak, Laura Wyatt, Hardayal Singh, Nadia Islam

**Affiliations:** 1 Department of Epidemiology and Health Promotion, New York University College of Dentistry, 433 First Avenue, 7th Floor, Room 726, New York, NY 10010 USA; 2 UNITED SIKHS, New York, NY USA; 3 Department of Population Health, New York University School of Medicine, New York, NY USA

**Keywords:** Community-based participatory research, Oral health equity, Implementation science, Consolidated Framework for Implementation Research, Asian American health, Community educators

## Abstract

The *Sikh American Families Oral Health Promotion Program* used a community-based participatory approach to develop, implement, evaluate, and disseminate a culturally tailored oral health/healthy living curriculum for the Sikh—South Asian community. Here, we examine the impact of community engagement throughout the process of program implementation in five Gurdwaras (places of worship) in New York and New Jersey and dissemination of the findings through targeted venues and the curriculum via e-Health resources. An interactive curriculum was developed (consisting of four core and three special topics) based upon a community-led needs assessment, adaptation of evidence-based oral health curricula, guidance from professional dental and medical associations, and input from Community Advisory Board (CAB) members. The Consolidated Framework for Implementation Research guided a mixed-method evaluation, consisting of both process and outcome measures. Five trained community educators delivered a total of 42 educational sessions. Improved oral hygiene behaviors and self-efficacy were found among program participants. For participants with no dental insurance prior to program enrollment (*n* = 58), 81.0% credited the program with helping them obtain insurance for themselves or their children. Further, for participants with no dentist prior to program enrollment (*n* = 68), 92.6% credited the program with helping them or their children find a local dentist. Short videos in Punjabi were created in response to feedback received from community educators and CAB members to reach men, especially. Community engagement was key to successful program implementation and dissemination, from the implementation leaders (community educators) to the opinion leaders and champions (CAB members).

## Introduction

The inaugural US Surgeon General’s report on oral health, released in May 2000, drew national attention to the burden of oral diseases and conditions that is disproportionately borne by impoverished communities and racial/ethnic minority populations at each stage of the life course [1]. Poor nutrition, lack of preventive oral health care, violence leading to face trauma, and tobacco and alcohol use harm teeth and their supporting structures, leading to dental caries (beginning in early childhood and continuing throughout life), periodontal diseases and tooth loss (especially in adults), and oral and pharyngeal cancers (formerly disorders of older adults, but increasingly affecting younger men, especially, due to oral human papillomavirus genotype 16 infection) [2, 3].

Social justice, meaning fairness and equity, has been invoked as the foundation of public health [4]. Community-based participatory research (CBPR) is consonant with this vision, since it may be usefully viewed as a partnership approach that facilitates capacity building and policy change through equitable engagement of diverse partners [5]. A corollary is that CBPR may be considered a translational strategy for diverse communities to improve health equity [6]. Our particular focus in this article is on dental justice [7]. We view oral health as the measure of a just society [8] and improved access to oral health care as essential to promoting overall health and well-being for vulnerable and underserved populations [9].

### Sikh American Families Oral Health Promotion Program

In response to a community needs and resources assessment completed in 2010 that identified access to oral health care as a priority in the community, the *Sikh American Families Oral Health Promotion Project* team sought and received funding in August 2012 from the DentaQuest Foundation [10] to address oral health promotion in the Sikh American community and build capacity for community engagement around oral health [11, 12]. UNITED SIKHS, the lead partner, was founded in 1999 to assist in the socioeconomic development of immigrant communities in Queens, New York [13]. It currently has chapters in America, Asia, and Europe that pursue projects for the spiritual, social, and economic empowerment of underprivileged and minority communities [13]. Its project partners include the New York University (NYU)—City University of New York (CUNY) Prevention Research Center [14], the NYU College of Dentistry [15], and Gurdwaras (Sikh places of worship) in New York and New Jersey [11, 12].

Our initial review of the scientific literature found that having a regular source of care and having dental insurance were important predictors of utilization of oral health care services among immigrants in New York City [16]. On the other hand, elevated oral cancer risk [17] and associated behaviors such as areca nut and betel quid chewing among South Asian immigrants [18] did not apply to the Sikh American community, due to explicit prohibitions against tobacco, alcohol, and drug use [19]. Instead, Sikhism preaches a message of devotion and remembrance of God at all times, truthful living, equality, and social justice, while denouncing superstitions and blind rituals [19]. The evidence base emphasized a need for more studies to elucidate the complex relationships of ethnicity, socioeconomic status, and culturally influenced factors that impact the oral health of immigrants [20].

Accordingly, the project began with a descriptive study to ascertain the prevalence of oral health and general health conditions in local Sikh American communities, barriers to access of dental care, and assets consisting of language- and culturally appropriate oral health resources [11]. Findings of clinical assessments conducted for 177 Sikh adults were that more than half had at least one decayed tooth, and again more than half had at least one missing tooth [11]. Focus group results conducted with Sikh adults confirmed that lack of dental insurance was a major concern, as was difficulty finding a local dentist whom they liked [11]. Full results are available from the authors upon request. A mixed methods approach was employed, incorporating both qualitative and quantitative methods and integration of multiple methods to increase depth of understanding while improving reliability and validity of findings [21, 22]. Questions were adapted to ensure cultural relevance to the Sikh American community and were reviewed by Community Advisory Board (CAB) members (composed of representatives of a diabetes-prevention community coalition, faculty and students from New York University, community-based oral health care providers, and leaders from local faith-based organizations, including South Asian members who speak Punjabi) to ensure cultural appropriateness [12]. At their request, oral health knowledge questions were included in the survey to gain a baseline understanding of oral health information within the Sikh American community [11].

### Consolidated Framework for Implementation Research

The conceptual framework informing this project is the Consolidated Framework for Implementation Research (CFIR) [23]. A CFIR technical assistance website is available for individuals considering using the CFIR to evaluate an implementation or design an implementation study [24]. The CFIR provides a menu of constructs that have been associated with effective implementation and have been used in a range of applications [24].

**Fig. 1:**
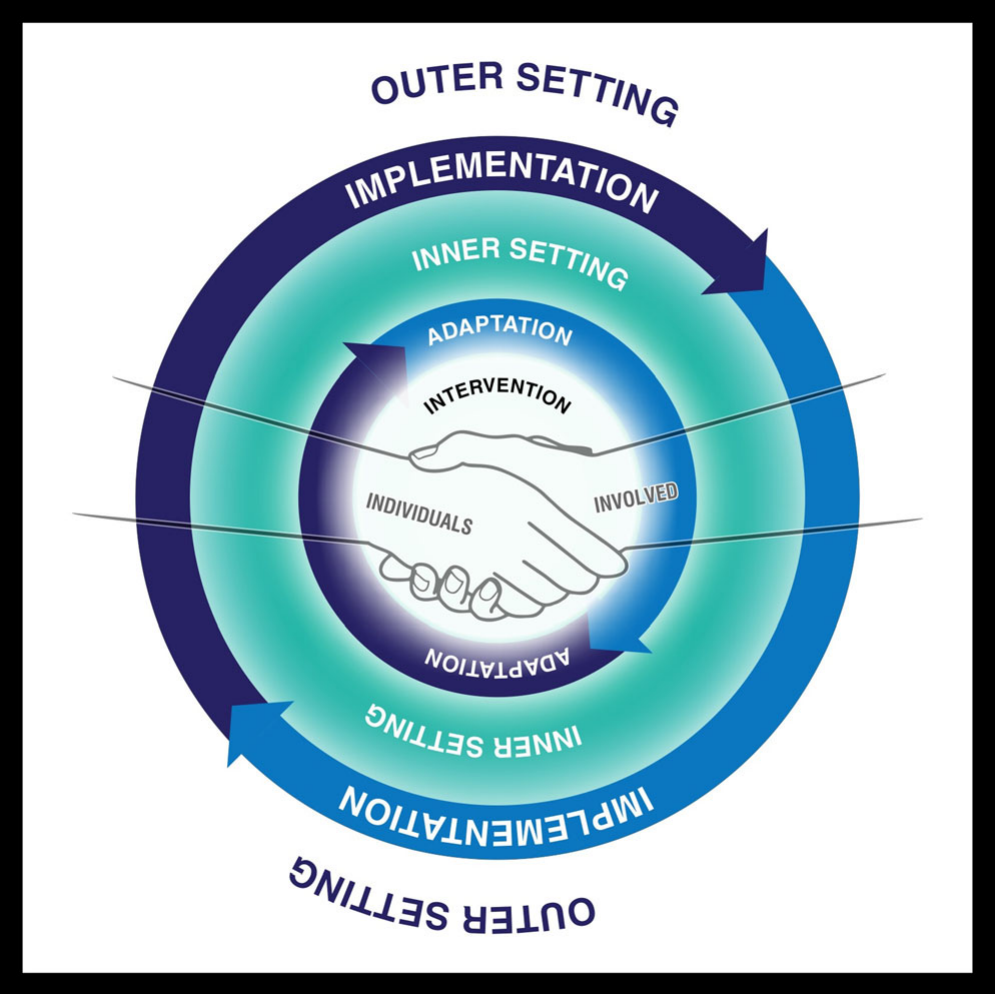
The five major domains of the Consolidated Framework for Implementation Research: the intervention, the inner setting, the outer setting, the individuals involved, and the process by which implementation is accomplished. Adapted from [23]. First appeared in [25]. Printed with permission

Figure 1 presents a stylized version of the five major domains of the CFIR (the intervention, the inner setting, the outer setting, the individuals involved, and the process by which implementation is accomplished) that has proven useful in prior oral health research [25–27].

Guided by the CFIR and the descriptive study completed in Year 1 of the project, the primary goals of the *Sikh American Families Oral Health Promotion Program* were to (1) develop and implement an intervention among Sikh Americans that promotes oral health to be delivered by trained community educators in local Gurdwaras and through e-Health modalities and (2) build closer relationships with local oral health care providers to improve access to and utilization of culturally relevant and affordable oral health care for the Sikh American community.

## Methods

### Community engagement around the design of the intervention

Utilizing an integrated CBPR—CFIR approach, the project partners developed an oral health promotion/healthy living curriculum for the Sikh—South Asian community based on the following five key recommendations derived from the analysis of the descriptive study in Year 1.

(1) Implement a linguistically and culturally tailored oral health promotion program for a largely Punjabi-speaking Sikh community utilizing community educators and peer-to-peer models.

(2) Provide information and services to link participants and their children to Medicaid, health benefits exchanges, and health and dental insurance plans, since health and dental insurance rates were found to be low, and cost was the primary reason cited for foregoing dental care.

(3) Facilitate improved access to and receipt of quality oral health care services and formalize relationships with local dental providers, as the majority of participants did not have a regular dentist or visit a dentist in the last year, and many focus group participants reported negative experiences with past dental care.

(4) Promote oral hygiene behaviors by demonstrating proper brushing and flossing techniques across the life span through a series of interactive, practical workshops, and utilize e-Health modalities to supplement in-person trainings for community members with access to technology.

(5) Encourage healthy lifestyles, including evidence-based, culturally tailored nutrition and physical activity guidelines, to address the high prevalence of overweight/obesity and hypertension and low levels of physical activity in the Sikh American community.

Research staff identified existing oral health curricula and authoritative guidelines from dental and medical associations for review and input from CAB members. The developed curriculum included 4 core modules on (1) oral health care, (2) oral health and nutrition, (3) oral health and chronic disease, and (4) oral health care access, along with three special interest topics devoted to oral health and health care for (1) women, infants, and young children, (2) adolescents, and (3) older adults [11]. Project partners sought to incorporate interactivity throughout the sessions to encourage participant engagement and motivation, such as hands-on demonstrations and models, games, and other adult learning techniques.

### Recruitment and training of community educators

The project was directed by one of the authors (R.K.), a Sikh community health worker supervisor. Nine Sikh community members from each of the six implementation sites (Gurdwaras) were recruited by CAB members from the affiliated congregations and trained as community educators to deliver the intervention (oral health promotion workshops) at each Gurdwara (inner setting).

**Fig. 2:**
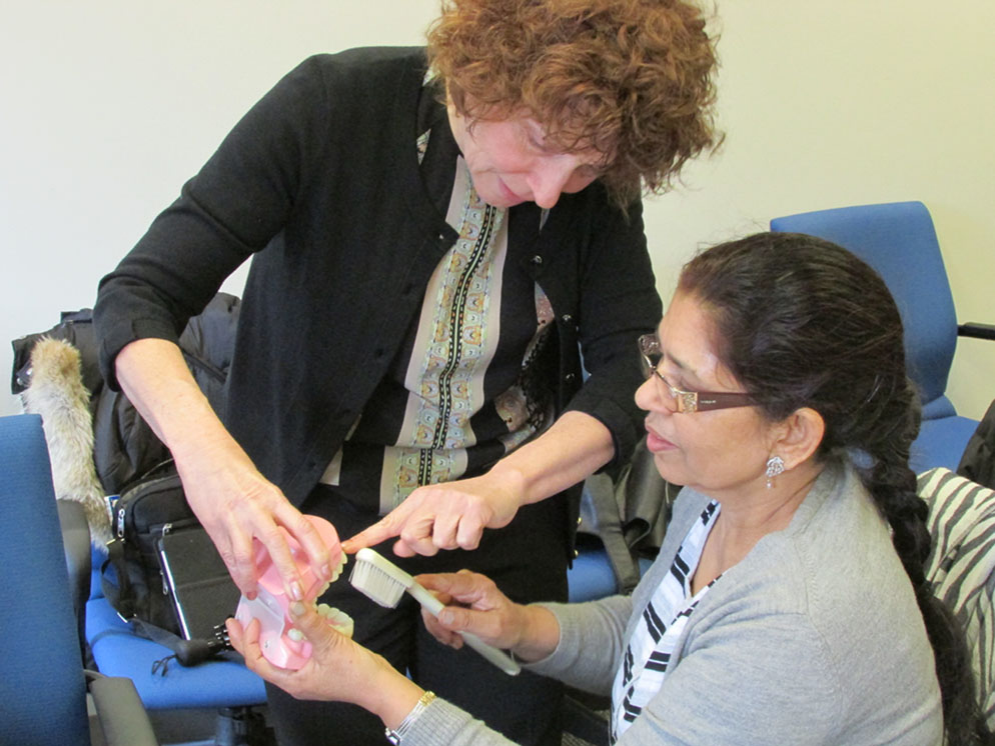
A dental hygiene faculty member demonstrates proper tooth brushing with fluoride toothpaste technique to a Sikh community educator using a model

Project partners, including dentists, dental hygienists, and research staff, developed and participated in a 4-day community educator training. Hands-on instruction was provided on proper brushing and flossing techniques, culturally tailored health promotion methods (i.e., preparing healthy Sikh meals and using the plate method to determine the proper balance and size of portions), and goal-setting skills. Trainees then demonstrated the presented procedures back to the trainers using models (see Fig. 2).

At the end of the training, the community educators collaborated in groups to practice delivering short excerpts from the curriculum to their peers, with the trainers providing comments and assistance. The community educators all spoke Punjabi and were members of the Gurdwaras where they delivered the intervention.

### Implementation of the intervention

Community educators and CAB members recruited workshop participants from the congregations of the six targeted Gurdwaras. A pilot round was implemented in Spring 2014 at four Gurdwaras and program refinements were made based upon community educator and participant feedback. The program was subsequently implemented in five Gurdwaras over 2 years, with workshops held every other week. Due to difficulties in coordinating schedules between the community educator and her participants, only one session was conducted at the sixth Gurdwara.

Workshop sessions were scheduled to last 30–45 min. In practice, however, they lasted an average of 60–75 min, since new participants joined sessions in progress and community educators were willing to repeat information and demonstrations for their benefit. By scheduling workshops to dovetail with ongoing Gurdwara activities such as Khalsa school (Sunday school) and weekly prayer groups, it was possible to engage more participants in the oral health promotion program.

### Program evaluation

The project team evaluated the intervention using both process and outcome measures. Discussions were held regularly with the community educators to understand more about their challenges and facilitators in implementing the program. Implementation fidelity, that is, the degree to which the community educators delivered the workshops as intended, was assessed using self-report and observations by the research staff [28]. De-briefing meetings were held after each session where any noted problems or challenges with intervention fidelity were discussed and resolved.

The data presented here are based upon a survey that was developed by the project team and reviewed by CAB members to ensure cultural appropriateness. Participants completed the survey 1 month after the final workshop session they attended, which included both the pre- (before the intervention) and post-test (after the intervention) responses. Changes in behavior and self-efficacy due to the intervention were measured using a retrospective pre-post design [29], in which participants were first asked, “Prior to beginning the program …” followed by, “At the present time …” Program satisfaction was measured by asking participants if they “strongly agree, agree, disagree, or strongly disagree” with statements about the community educators, the information presented in the workshops, whether the intervention helped change behaviors related to nutrition and exercise, and whether the intervention helped them or their children to obtain dental insurance or find a local dentist. Finally, barriers to full participation in the intervention were identified as well as overall satisfaction with the intervention and comments regarding what the participants liked, did not like, and might be improved in the program.

All items were summarized using descriptive statistics [30]. Differences in the retrospective pre- and post-test measures were compared using the Chi-square test [30]. All statistical analyses were performed using SPSS software, version 21.0 (IBM Corporation, Armonk, New York USA). A two-sided *P* value of <0.05 was considered statistically significant.

## Results

Fully 96% of the participants in the *Sikh American Families Oral Health Promotion Program* were born in India, with a mean age of 49.5 years (standard deviation = 13.1). More than half of the participants (55%) were women, only one-sixth (17%) spoke English at home, and one-third (33%) reported earning less than a high school education. At the beginning of the intervention, 29% of the participants reported having health insurance, 35% reported having a regular dentist, and one-quarter (24%) reported that there was a time in the last year when they needed dental care and could not get it. The study population is representative of adults from recent immigrant Sikh communities who attend Gurdwaras in New York and New Jersey.

The frequency of self-reported oral health promotion behaviors and self-efficacy regarding health-related activities of participants before and after attending the oral health promotion program are presented in Table 1.

**Table 1:** Frequency of self-reported behaviors and self-efficacy regarding health-related activities for participants in the *Sikh American Families Oral Health Promotion Program* when asked to think back to the time prior to attending the first workshop and at the present time after having attended the workshops (*n* = 126)

	Prior to beginning the program (attending the first workshop)	At the present time (after having attended the workshops)	*P* value^a^
	% (*n*)	% (*n*)	
Percent endorsing the desired oral health promotion behavior			
Brushes teeth more than once a day for at least 2 min	12.8 (16)	69.0 (87)	<0.001
Flosses at least once a day	7.9 (10)	42.0 (53)	<0.001
Percent endorsing the very confident option			
Feels that knows how to take good care of mouth, teeth, and gums	0	57.3 (71)	<0.001
Feels that are able to take good care of mouth, teeth, and gums	3.2 (4)	72.4 (89)	<0.001
Feels that are able to eat a healthy diet	4.8 (6)	65.0 (80)	<0.001
Feels that are able to be physically active for 150 min/week	6.3 (8)	66.4 (83)	<0.001
Feels able to ask dentist or oral hygienist questions	4.8 (6)	61.6 (77)	<0.001

Denominators may differ due to missing values

^a^ *P* values correspond to the testing of differences between prior and present-time endorsements using the Chi-square test

Statistically significant improvements were found for participants after attending the program regarding both brushing teeth more than once a day for at least 2 minutes and flossing at least once a day. Moreover, statistically significant improvements were found for participants who were very confident in both knowing how and feeling able to take good care of mouth, teeth, and gums and feeling able to ask the dentist or dental hygienist questions. Similar findings were found for improvements in eating a healthy diet and being physically active for 150 min/week.

Upon completing the program, all of the participants strongly agreed or agreed that the community educators answered their concerns and questions and helped them to improve how they took care of their health (Table 2).

**Table 2:** Self-reported satisfaction with and assessment of the *Sikh American Families Oral Health Promotion Program* among participants 1 month after having attended the workshops (*n* = 126)

Program component	Percent
Percent who strongly agreed or agreed with the following statements	
The community health educator(s) answered my concerns and questions	100 (126/126)
The community health educator(s) helped me to improve how I take care of my health	100 (126/126)
The information and topics presented in the workshops were informative	100 (126/126)
The in-person demonstrations of how to brush and floss properly helped me to improve my oral health	100 (125/125)
The videos and tutorials available online helped me to improve my oral health (full intervention only)	98.5 (67/68)
The program helped me to eat a more healthy diet	100 (126/126)
The program helped me to increase my level of physical activity each week	98.4 (124/126)
Percent who affirmed the following oral healthcare characteristics	
Had dental insurance before program enrollment	53.6 (67/125)
Program helped to get dental insurance for self or children among those with no dental insurance	81.0 (47/58)
Had a dentist before program enrollment	45.6 (57/125)
Program helped to find a local dentist for self or children among those with no dentist	92.6 (63/68)
Percent who endorsed the following barriers to participating in workshops or other program activities	
Did not have transportation to sessions	10.3 (13/126)
Family obligations	30.2 (38/126)
Lack of child care	15.9 (20/126)
Work schedule conflicted with the sessions	19.0 (24/126)
Lack of interest	11.9 (15/126)
Session location was not convenient for me	2.4 (3/126)
Travel to home country conflicted with sessions	0.8 (1/126)
Percent who endorsed the following program satisfaction and sustainability items	
Self-reported being very satisfied or higher with the program (≥8 on a scale of 0–10)	95.0 (115/121)
Self-reported willingness to serve as a volunteer for the program in the future	52.3 (57/109)
Felt that the variety of workshop topics was just right	100 (122/122)
Felt that the length of time of each workshop was just right	76.6 (93/122)
Percent who felt that the length of time between workshops should be	
1 week	30.7 (36/122)
2 weeks	67.7 (84/122)
3 weeks	1.6 (2/122)

Denominators may differ due to missing values, questions that were asked in the full intervention only, or varying numbers of participants who were eligible to respond

Similarly, all of the participants believed that the information and topics presented in the workshops was informative and that the in-person demonstrations of how to brush with fluoridated toothpaste and floss properly helped them to improve their oral health. While 53.6% of participants reported having dental insurance before program enrollment, of the remaining 58 participants, 81.0% credited the program with helping them get dental insurance for themselves or their children. Likewise, while 45.6% of participants reported having a dentist before program enrollment, of the remaining 68 participants, 92.6% credited the program with helping them to find a local dentist for themselves or their children. Barriers that prevented Gurdwara congregants from participating in workshops or other program activities included lack of transportation (10.3%), family obligations (30.2%), lack of child care (15.9%), work schedule conflicts (19.0%), and lack of interest (11.9%). Overall, 95% of participants reported being at least very satisfied with the program (8 or higher on a 10-point scale).

When participants were asked what they liked about the program, they responded:

“First, sometimes I missed my appointment. Now I’m very concerned about my oral health [and] brush my teeth properly. I liked [learning] how oral health is important.”

“How by small efforts we can enjoy our teeth for a long lifetime.”

“Liked the most how to floss [and] plate method and also liked the workshops in Punjabi.”

“How to take care of dental and overall health. The way of teaching [through] demonstration and [the] friendly atmosphere. How to floss and brush my teeth was wonderful.”

“Whatever we [didn’t] learn from our doctors [was] because we never asked questions to doctors before.”

When participants were asked what they did not like about the program, they mentioned the following concerns:

“I need more classes and materials in my language. I cannot read English.”

“Maybe lesson should be a little short[er].”

Further, when asked what changes might improve the program, they advised:

“More about health dental cards and how to obtain them.”

“Have pictures scaring people if not taking care of teeth good.”

“Have more for kids.”

“Give us more handouts materials regarding nutritional topics.”

Other participant comments included:

“Good true community service. People mostly ignore dentist appointments and brush their teeth in [a] hurry, not properly.”

“The program helped me and my family to enjoy healthy life and be more active.”

“Program was very helpful. Way of demonstration teaching was good. It will save our health and money.”

“In future more programs can help our community to prevent diseases.”

## Discussion

The *Sikh American Families Oral Health Promotion Project* (the intervention) increased awareness of the importance of and knowledge about oral health, promoted evidence-based oral hygiene and healthy living activities, and improved access to dental care through increased dental insurance coverage and linkage to local dental providers for participants and their children. As in other impoverished communities [31], problems with teeth and gums were prevalent in the Sikh American communities in New York and New Jersey, even as oral health knowledge was low and barriers to obtaining oral health care were high [11]. In particular, the brushing with fluoride toothpaste and flossing demonstrations as part of the workshops delivered in Gurdwaras (the inner setting) were highly valued by the program participants, emphasizing the importance of hands-on, face-to-face health promotion support delivered by trusted community educators (the individuals involved).

Several problems arose while implementing the pilot intervention, which the project partners and community educators worked collaboratively to address for the subsequent iterations of the program. Major challenges included difficulties in recruiting participants, especially men, due to scheduling conflicts and lack of understanding among the congregants of the need for the oral health promotion program. As a result, additional sessions were offered to dovetail with other scheduled Gurdwara activities and both community educators and CAB members promoted the program to their members by emphasizing the importance of oral health to general health and well-being. Limitations of this research include the retrospective assessment, the potential for bias associated with self-reported behaviors, and whether or not any improved behaviors and access to care would lead to enhanced oral and general health outcomes.

On 15 August 2016, UNITED SIKHS launched via YouTube a series of short (2–4 minutes) videos in Punjabi delivered by a Sikh dental hygienist that are based upon input received from community educators and CAB members (see the announcement at http://unitedsikhs.org/PressReleases/PRSRLS-15-08-2016_1.html). The oral health video series consists of the following videos: (1) brushing (with fluoride toothpaste) tips and techniques; (2) flossing tips and techniques; (3) plate planner (eating right with the plate method); and (4) what to expect at your first dentist visit (see https://www.youtube.com/playlist?list=PLmXVZUNGmD0x7GLxPAT2hsJ19dkNvfJid). The affiliated Gurdwaras and New York and New Jersey state and regional oral health coalitions will help to disseminate this series to reach community members who have access to technology and whose work and family responsibilities preclude their attendance at workshops.

### Community engagement throughout the process of implementation and dissemination

As others have argued, behavior change leading to improved oral health is more likely to occur in settings where people feel more comfortable and open to receiving information, when messages are delivered by trusted members of their community, and when information is repeated multiple times by multiple people [32]. Hence, the program involved community members in the planning of the intervention, the recruitment of appropriate individuals, carrying out the planned activities, and reflecting on the process and outcome measures, in concert with the CFIR process constructs [23, 24] and previous research on community engagement to enhance Asian American health [33, 34]. Specifically, CAB members served as opinion leaders, community educators served as implementation leaders, and Gurdwara leaders served as program champions [23, 24].

### External policies that abetted the program (outer setting)

The single most important policy that abetted the program was the passage of the Affordable Care Act (ACA), which was signed into law by President Obama in 2010 and implemented in stages in subsequent years [35]. Dental coverage for children is now an essential health benefit [35]. Further, Medicaid expansion in New York and New Jersey means that more adults are now eligible to receive dental benefits [36].

Both before and after the program was funded, UNITED SIKHS built its organizational capacity by engaging in community health worker initiatives, implementing policies to increase access to healthy foods, and training navigator assistors to facilitate enrollment of community members into insurance programs. Thus, it was well positioned to assist program participants who lacked dental coverage in obtaining dental insurance [13]. Moreover, the NYU College of Dentistry is the largest Medicaid provider in New York State and a partner in this project, providing dental care access when other dental providers fail to accept Medicaid as a form of payment [15]. The program was able to leverage this partnership to identify local dental providers for program participants and their children, dentists and dental hygienists to assist in the clinical examinations as part of the needs assessment and the 4-day, in-person training for the community educators, and dental supplies as gifts to the workshop participants (e.g., fluoride toothpaste, soft-bristled toothbrushes, and dental floss). Importantly, the NYU-CUNY Prevention Research Center provided the research infrastructure to abet Institutional Review Board approval, evaluation design and analysis, and linkage to ongoing diabetes and hypertension prevention projects and research experts [14].

### Integrating community engagement and implementation science to achieve health equity

We believe that integrating community engagement and implementation science hold promise not only for improving health research [37, 38] but also for achieving health equity. Adapting evidence-based interventions to racial/ethnic minority populations with the active engagement of affected communities is one path forward [39]. Other approaches hold value, including incorporating theories from social and behavioral science [40] and utilizing systems thinking and modeling (see, e.g., [41]). Research projects that integrate CBPR and informatics have achieved success in engaging underserved populations and designing patient-facing technology [42]. By reconnecting the mouth to the rest of the body in public health research, practice, and policy [43] and ensuring culturally and linguistically relevant services to communities regardless of race/ethnicity, socioeconomic position, age, gender, sexuality, or immigration status, we move closer to the Sikh ideal of recognizing the human race as one [13].

## Abbreviations

ACA: Affordable Care Act
CAB: Community Advisory Board
CBPR: Community-based participatory research
CFIR: Consolidated Framework for Implementation Research
CUNY: City University of New York
HIPAA: Health Insurance Portability and Accountability Act
IRB: Institutional Review Board
NYU: New York University
